# Three-Dimensional Structure of N-Terminal Domain of DnaB Helicase and Helicase-Primase Interactions in *Helicobacter pylori*

**DOI:** 10.1371/journal.pone.0007515

**Published:** 2009-10-20

**Authors:** Tara Kashav, Ramgopal Nitharwal, S. Arif Abdulrehman, Azat Gabdoulkhakov, Wolfram Saenger, Suman Kumar Dhar, Samudrala Gourinath

**Affiliations:** 1 School of Life Sciences, Jawaharlal Nehru University, New Delhi, India; 2 Special Centre of Molecular Medicine, Jawaharlal Nehru University, New Delhi, India; 3 Institut für Chemie und Biochemie/Kristallographie, Freie Universität Berlin, Berlin, Germany; University of Hyderabad, India

## Abstract

Replication initiation is a crucial step in genome duplication and homohexameric DnaB helicase plays a central role in the replication initiation process by unwinding the duplex DNA and interacting with several other proteins during the process of replication. N-terminal domain of DnaB is critical for helicase activity and for DnaG primase interactions. We present here the crystal structure of the N-terminal domain (NTD) of *H. pylori* DnaB (*Hp*DnaB) helicase at 2.2 Å resolution and compare the structural differences among helicases and correlate with the functional differences. The structural details of NTD suggest that the linker region between NTD and C-terminal helicase domain plays a vital role in accurate assembly of NTD dimers. The sequence analysis of the linker regions from several helicases reveals that they should form four helix bundles. We also report the characterization of *H. pylori* DnaG primase and study the helicase-primase interactions, where *Hp*DnaG primase stimulates DNA unwinding activity of *Hp*DnaB suggesting presence of helicase-primase cohort at the replication fork. The protein-protein interaction study of C-terminal domain of primase and different deletion constructs of helicase suggests that linker is essential for proper conformation of NTD to interact strongly with *Hp*DnaG. The surface charge distribution on the primase binding surface of NTDs of various helicases suggests that DnaB-DnaG interaction and stability of the complex is most probably charge dependent. Structure of the linker and helicase-primase interactions indicate that *Hp*DnaB differs greatly from *E.coli* DnaB despite both belong to gram negative bacteria.

## Introduction

*Helicobacter pylori (H. pylori)* is a gram-negative, microaerophilic spiral shaped bacterium that infects more than 50% of the human population globally and is responsible for causing chronic gastritis, peptic ulcer and gastric cancer [1]. Replication system in *H. pylori* has unique features compared to other well studied microorganisms like *E. coli* and *Bacillus subtilis* such as the absence of the *recF* gene, the presence of the *dnaA* gene ∼600 kb away from the *dnaN*–*gyrB* genes and the absence of the *dnaC* gene [2].

Helicases are nucleic acid motor proteins that derive energy from NTP hydrolysis and unwind DNA in 5′ to 3′ direction [3]. In *E. coli*, DnaB helicase plays a central role; which displays protein-protein interactions right from the beginning to the end of replication process. The partners of DnaB include but not limited to DnaC (loading partner), DnaA (initiator) [4], DnaG (primase) [5], SSBs (Single Strand Binding proteins) [6], τ-subunit of DNA polymerase [7], and replication termination protein [8]. The loading partner DnaC recruits DnaB as DnaB_6-_DnaC_6_ complex at *ori*C site and plays an important role during replisomal complex formation [9], [10]. Interestingly, bioinformatics studies shows the absence of a DnaC homolog in the *H. pylori* genome [2], [11] and yeast two-hybrid studies shows that *H. pylori* DnaB (*Hp*DnaB) does not interact with *Hp*DnaA [12]. Our biochemical studies earlier had confirmed that *Hp*DnaB is able to complement the DnaC function and loads itself to the *ori*C site to form the pre-replication complex [13].

Helicases are classified into three super families (SF1, SF2 and SF3) and one small family (F4) based on the presence of specific motifs [14]. The *Hp*DnaB belongs to family 4 that consists of homohexameric replicative helicases. Each monomer consists of a N-terminal domain linked via a flexible linker to the C-terminal domain. The C-terminal domain is highly conserved as all the five conserved motifs including the ATPase motif and DNA binding motif are located within this region. The N-terminal region is highly variable among the helicases, similar to its binding partner, the C-terminus of DnaG primase.

Electron microscopic studies of the full length structure of DnaB helicase from *E. coli* (*Ec*DnaB) [15], RSF1010 helicase [16], *Bacillus subtilis* bacteriophage SPP1 helicase (G40P) [17] and bacteriophage T7 gene 4 DnaB [18] reveal a closed ring hexameric state with several different quaternary states like 6-fold rotational symmetry (C_6_) or 3-fold rotational symmetry (C_3_) or an intermediate between the two (C_3_ C_6_). Very recently the crystal structures of *Bacillus subtilis* bacteriophage SPP1 G40P helicase (G40P) [19], *Bacillus stearothermophilus* DnaB (*Bst*DnaB) [20], *Thermus aquaticus* DnaB (*Taq*DnaB) [21] and the N-terminal domain structure of *Mycobacterium tuberculosis* DnaB (*Mtb*DnaB) [22] have also been reported. The crystal structures have illustrated that the linker region between the globular N-terminal domain and the C-terminal domain consists of three α-helices, where first two helices forms α-hairpin structure. The structure of *Bst*DnaB in complex with primase C-terminal domain, along with the structural and mutational studies on G40P helicase revealed that the N-terminus of helicases plays a crucial role in binding to primase and triggers primase mediated enhancement of helicase activity [19], [20].

Our recent biochemical findings suggest that the N-terminus and linker region plays an important role in the multimerisation, quaternary state transition and activity of *Hp*DnaB [23]. The DelN1*Hp*DnaB deletion mutant (1- 66 residues deleted), which lacks the NTD but consists of an intact α-helical hairpin and the DelN2*Hp*DnaB deletion mutant(1–133 residues deleted), which lacks the first helix from α-helical hairpin and the NTD show 20% and 30% reduction in helicase activity respectively. The electron microscopic studies emphasized the essential role of N-terminal region for the quaternary state polymorphism in *Hp*DnaB [23]. Similarly, the N-terminal domain appears to be critical in *Ec*DnaB, *Bst*DnaB and G40P helicase since an equivalent NTD deletion mutants consisting of the C-terminal helicase domain and a part of the linker region exhibit *in vitro* ATPase activity but fails to demonstrate any helicase activity [24]–[26]. However, the importance of NTD and linker for helicase activity does not explain the function they play during DNA unwinding process at the replication fork.

Replicative helicases bind DnaG primase to synchronize DNA unwinding with RNA primer synthesis during the process of replication [5]. DnaG consists of an N-terminal Zinc binding domain (ZBD), RNA polymerase domain (RPD) and a C-terminal Helicase binding domain (HBD). In different organisms, the DnaB-DnaG complex stability varies remarkably. In *E. coli*, the interaction is relatively weak [27] whereas in *B. stearothermophilus,* the DnaB-DnaG complex is highly stable as can be purified intact by gel filtration [24]. Based on these observations, we were interested in studying the DnaB-DnaG interactions in *H. pylori*.

Taking leads from the homology modeling together with *in vitro* and *in vivo* function of different deletion mutants of *Hp*DnaB as shown earlier [23], we defined the N-terminal and C-terminal domains separated by the flexible linker region. We further cloned deletion constructs of N-terminal domain (NTD) consisting of 1 to 121 residues and N-terminal domain with longer linker consisting of 1 to 144 residues (NTDL). To understand the roles played by NTD in helicase function, the importance of the linker region and helicase primase interactions, we present here the structure of NTD of *Hp*DnaB at 2.2 Å resolution and compare it with other NTD structures. Further, we were interested to see whether helicase-primase interaction is conserved in slow-growing pathogenic bacteria like *H. pylori* and whether it can account for the stimulation of key functions of these proteins at the replication fork. We report here the cloning, expression and purification of *H. pylori* primase (*Hp*DnaG) and C-terminal domain (CTD) of *Hp*DnaG primase (*Hp*DnaG) for the very first time. We had investigated the role of *Hp*DnaG as a possible binding partner of *Hp*DnaB and its effect on helicase activity.

## Materials and Methods

### Cloning, expression and purification of NTD of *Hp*DnaB Helicase

The gene corresponding to NTD of *Hp*DnaB construct was amplified by Polymerase Chain Reaction (PCR) using wt*Hp*DnaB construct as a template. The PCR amplification of 1–363 nucleotides was done using *Taq* DNA polymerase (MBI *Fermentas*) with the following forward and reverse oligonucleotide primers:

FP: 5′ CATGCCATGGATCATTTAAAGCATTTGC 3′ and

RP: 5′ CCGCTCGAGGGCTTGCTCTCTAATGG 3′

The amplified DNA was inserted into NcoI/XhoI digested expression plasmid vector pET 28a (Novagen, Madison, WI, USA) with C-terminal six residue His tag.

A little longer construct of NTD (NTDL) of *Hp*DnaB Helicase was made to include helical hair pin region. The PCR amplification of 1–144 amino acids was done using *Taq* DNA polymerase (MBI *Fermentas*) with the forward and reverse oligonucleotide primers, FP: 5′ CATGCCATGGATCATTTAAAGCATTTGC 3′ and

RP: 5′ CCGCTCGAGGCCATTCAATAACGCATAG 3′

The amplified DNA was inserted into NcoI/XhoI digested expression plasmid vector pET 28a (Novagen, Madison, WI, USA) with C-terminal six residue His tag.

**Figure 1:**
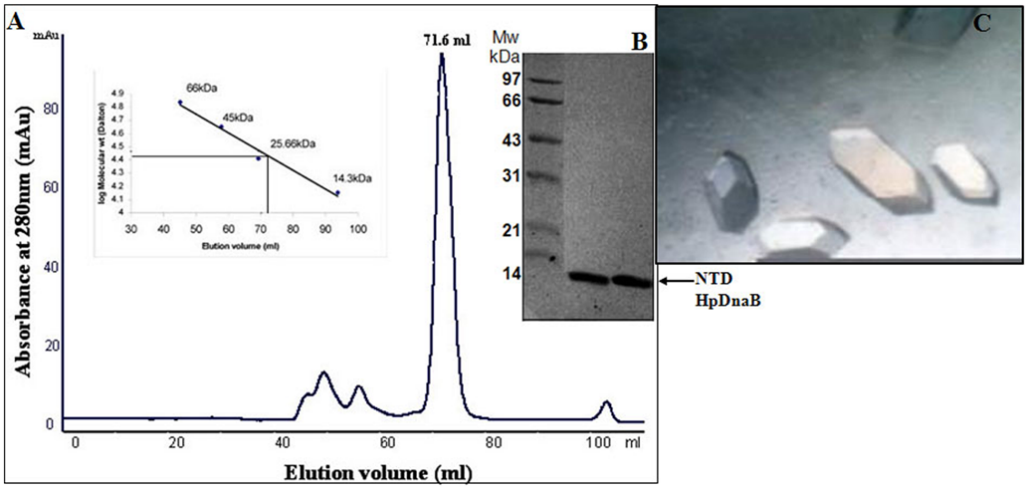
Purification and crystallization. (A) A gel filtration chromatogram of NTD of *Hp*DnaB as obtained by passing through Hi Load superdex-75 16/60 column. *INSET* shows the elution pattern of gel filtration standards plotted in logarithmic scale against the elution volume. Molecular mass of eluted dimeric form of NTD of *Hp*DnaB is deduced from the standard plot. (B) SDS-PAGE gel of purified NTD of *Hp*DnaB (lane1) along with protein marker (PM). (C) Rhombohedral shaped crystals of NTD of *Hp*DnaB obtained at 277K by hanging drop vapor diffusion method.

The recombinant plasmid containing the NTD of *Hp*DnaB insert was transformed into BL21 (DE3) cells. Freshly transformed BL21 cells were grown in 2% LB medium containing 50 µM kanamycin at 37°C to an O.D_600_ of 0.4. Then, the culture was induced with addition of 1 mM IPTG. Cell growth continued till O.D._600_ reached 0.9 and cells were centrifuged at 5000 rpm. The harvested cells were resuspended in lysis buffer containing 50 mM Tris.HCl pH 8.0, 0.3 M NaCl, 1 mM Imidazole, 100 µM Phenyl Methyl Sulphonyl Fluoride (PMSF) and lysed by addition of lysozyme (1 mg/ml) that was then incubated for ten minutes at 37°C. The clarified lysate was passed through a Ni-NTA column. The bound protein was eluted with 300 mM Imidazole (50 mM Tris, 150 mM NaCl, 0.3 M Imidazole, pH 7.5). The positive fractions were pooled, concentrated and purified further on a Hi Load superdex-75 16/60 column (GE) equilibrated with 20 mM Tris.Cl buffer pH 7.5 containing 100 mM NaCl, 1 mM EDTA, 5 mM βME (Figure 1A). The fractions were loaded on 10% SDS-PAGE (Figure 1B) and pure fractions were pooled and concentrated to 10 mg/ml in YM3K centricon tubes (Amicon). The concentrated protein was used for crystallization and biochemical assays. The full length *Hp*DnaB and DelN2*Hp*DnaB were expressed and purified as described before [23]. The expression and purification of NTDL was similar to NTD of *Hp*DnaB as mentioned above.

### Cloning, expression and purification of full length and CTD of *Hp*DnaG Primase

*H. pylori* genomic database shows an ORF, Hp0012 (strain 26695) annotated as DNA primase (DnaG). The 1.7 kb fragment of *Hp*DnaG was amplified using *H. pylori* genomic DNA (ORF Hp0012) as template and forward (5′-ACGGTCGACATGATTCTTAAAAGTTCCATTG-3′) and reverse (5′-ACGGTCGACATATGGCGACTAATTCTCCTTG -3′) primers having SalI restriction site and *Taq* DNA polymerase (5PRIME PCR Extender system, 5PRIME gmbH, Hamburg Germany).

The PCR product was subsequently digested with SalI restriction enzyme and cloned in pET30b (Novagen, Madison, WI, USA) expression vector at SalI site. *E.coli* strain BL21 (DE3) was transformed with pET30b-*HpDnaG* construct and grown at 37°C in LB medium containing 50 µM kanamycin until the O.D._600_ reached 0.6. Induction of His_6-_HpDnaG protein (*Hp*DnaG) was carried out using 1 mM IPTG at 22°C for 4 hrs. The bacterial cells were centrifuged to get the pellet, and the pellet was frozen in −80°C. Protein purification was carried out using Ni-NTA agarose beads (Qiagen, Hilden, Germany) according to manufacturer's instructions. The eluted protein was dialyzed against 20 mM MES, 100 mM NaCl, 10% glycerol, 1 mM EDTA, 100 µM PMSF and 10 mM βME. Second step of purification was carried out by ion- exchange chromatography using resource S ion-exchange column (GE Healthcare, Uppsala Sweden) in accordance with the manufacturer's instructions. The peak fractions were checked in 10% SDS-PAGE and dialyzed against 50 mM Tris.HCl pH 7.5, 100 mM NaCl, 10% glycerol, 100 µM PMSF, and 10 mM βME.

The CTD of DnaG Primase was amplified by using *Taq* DNA polymerase (MBI *Fermentas*) for PCR with genomic DNA of *H. pylori* (ORF Hp0012) as template. Following oligonucleotide primers were used as forward and reverse primers:

FP: 5′ CTAGCTAGCGAGCGAGTCTCTTTTCAGCCTTT 3′ and

RP: 5′CGGCTCGAGTATGGCGACTAATTCTCCTTGTTTT 3′

The amplified PCR product was digested with Nhe1/Xho1 and inserted into Nhe1/Xho1 digested expression plasmid vector pET21c (Novagen, Madison, WI, USA). The CTD of *Hp*DnaG with C-terminal six residues His tag was expressed and purified similar to the NTD of *Hp*DnaB.

### Crystallisation and data collection of NTD of *Hp*DnaB

The purified NTD and NTDL of *Hp*DnaB (10 mg/ml) was subjected to various crystallization screens (Hampton research) both at 16°C and 4°C. Hanging drops were prepared in 24-well plates by mixing 3 µl protein solutions (10 mg/m1) with 3 µl reservoir solution and were equilibrated against 500 µl reservoir solution. Crystalline precipitates were observed for NTD of *Hp*DnaB with 25% PEG 3350, 0.2 M Ammonium sulfate and 100 mM Bis-Tris pH 5.5 as precipitant during the initial screens. After optimization of the physico-chemical parameters affecting crystallization, the best conditions were obtained at 4°C using the hanging-drop vapor diffusion method (Figure 1C). So far, the crystallization trials on the NTDL were not successful. To provide cryo-protection, crystals of NTD of *Hp*DnaB were treated with mother liquor containing 30% PEG400. The X-ray diffraction experiments were performed at 100K from crystals mounted in cryoloops at cryogenic conditions and flash frozen in liquid nitrogen. The X-ray diffraction data were collected at BESSY synchrotron beam line at wavelength of 0.9184 Å. The data set were indexed and scaled with program HKL2000 [28] or XDS [29].

### Structure determination and refinement

**Figure 2:**
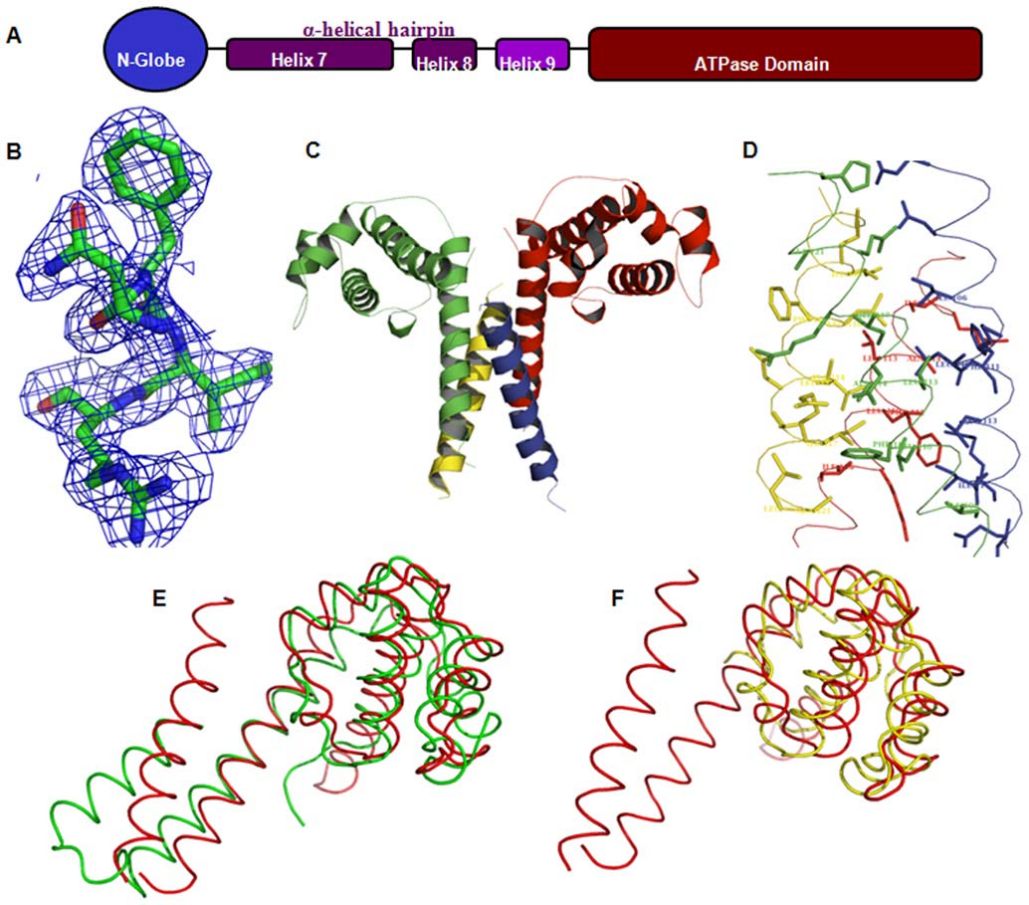
Schematic of *Hp*DnaB and structure of NTD of *Hp*DnaB helicase. (A) A schematic of *Hp*DnaB helicase showing N-globe separated by a linker from C-terminal ATPase domain. The linker consists of three helices, helix7, helix8 and helix 9. The NTD of *Hp*DnaB consists of N-globe and helix7 of linker; NTDL consists of N-globe, helix 7 and helix 8. (B) A portion of molecule showed with 2Fo-Fc electron density map at 2σ calculated at 2.2 Å resolution. (C) The overall structure of NTD of *Hp*DnaB consists of two complete monomers (red and green) and two short helices (blue and yellow) forming a four helix bundle at the dimeric interface is displayed as ribbon diagram. (D) The dimer-dimer interface consists of hydrophobic residues shown as sticks with the Cα backbone of the four helix bundle; color scheme is same as in figure 2C. (E) The superimposition of NTDs of *Hp*DnaB (red) and *Mtb*DnaB (green) shows that the fragmented helix in NTDs of *Hp*DnaB falls on the small helix of the helical hairpin of NTD of *Mtb*DnaB forming a helical hairpin like structure. (F) The superimposition of NTD of *Hp*DnaB (red) on NTD of *Ec*DnaB (yellow) demonstrates the apparent differences in the N-globe region as shown by the root-mean-square deviation (RMSD).

The structure of NTD of *Hp*DnaB was solved by molecular replacement using *Phaser* [30] with the structure of N-terminal domain of *Mycobacterium tuberculosis* (PDB id: 2r5u; Biswas et al, 2007) as a search model. For refinement, initially *Refmac5* [31] was used and further improvements were done by *CNS* [32]. Along with two molecules of NTD of *Hp*DnaB in one asymmetric unit, a large extra electron density was observed near the dimeric interface. This extra density showed well connected main chain and MALDI results (Figure S1) allowed us to build the side chains. The structure was improved with iterative model building using *COOT* graphics package [33], the final structure had very good electron density (Figure 2B). Water molecules were picked up both manually as well as with appropriate routines in CNS. The PyMOL program [34] was used to generate the figures. The R_factor and R_free values for this structure are little above the average values for the structures refined at given resolution [35], this could be because the crystals showed very high solvent content (77%) and some non traceable extra electron density, which could be due to low occupant degraded peptides.

### Helicase assay

**Figure 3:**
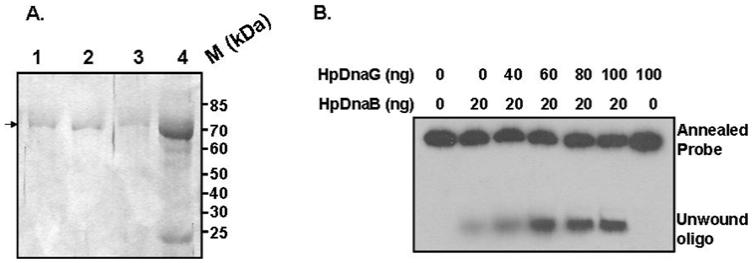
Purification of wt*Hp*DnaG and its effect on wt*Hp*DnaB helicase activity. (A) SDS-PAGE of His_6_ *Hp*DnaG. Lanes 1–3 show the peak fractions after mono S (ion exchange) purification and lane 4 shows the partially purified protein after Nickel affinity chromatography. (B) Stimulation of *Hp*DnaB helicase activity in the presence of *Hp*DnaG. Different amounts of *Hp*DnaG were added in *Hp*DnaB helicase reaction as mentioned in the materials and methods. The positions of the annealed probe and unwound oligos are shown. The results indicate that *Hp*DnaG stimulate *Hp*DnaB helicase activity.

Helicase assay substrate was prepared as described earlier by Sharma *et al* [36]. Helicase assay was carried out in 20 µl reaction mixture containing 20 mM Tris.HCl pH 8.0, 8.0 mM DTT, 2.5 mM MgCl_2_, 2.0 mM ATP, 80 µg/ml BSA, 10 mM KCl, 4% sucrose, 10 fmol of helicase substrate and the indicated amount of *HpD*naB and *HpD*naG proteins (Figure 3B). The reaction mixture was incubated at 37°C for 30 minutes. The reaction was stopped by addition of 5 µl 5X stop buffer (1.25% SDS, 75 mM EDTA, 25% glycerol) and loaded on 10% native PAGE to resolve the products. The gel was dried and exposed to X-ray film (Kodak) and developed subsequently.

### Surface Plasmon Resonance (SPR) experiments

All binding experiments were performed on a Biacore 2000 apparatus (Biacore, GE Healthcare) at 25°C in running buffer (10 mM Hepes pH 7.4, 150 mM NaCl, 3 mM EDTA, and 0.005% P20 surfactant). A total of 750 resonance units (RU) of CTD of *Hp*DnaG primase (ligand) were immobilized on a research grade CM4 chip (Biacore™) in 10 mM sodium acetate, pH 5.0 according to the manufacturer's amine coupling kit. The un-reacted residues on the surface were blocked by two washes of 1 M ethanolamine, pH 8.5. One flow cell left blank was used as reference. Helicase samples (10 µM) were prepared in running buffer and were injected at 20 µl/min across the sensor surface. Signal changes on the activated/blocked control panel were subtracted from the DnaG-DnaB binding interactions using in-line reference signal and the subtracted sensorgrams were analyzed. The surface was regenerated with regeneration buffer consisting of a pulse of 25% ethanol. This experiment was repeated for full length helicase *Hp*DnaB, DelN2*Hp*DnaB, NTDL and NTD of *Hp*DnaB. Data analysis was performed with the BIAevaluation software (Pharmacia, version 2.1). The dissociation and association rate constants *k*_diss_ and *k*_ass_ respectively were determined by direct curve fitting following the equation *R*_t_ = *R*_0_ e^−*k*^_diss_^(*t*−*t*0)^, where *R*_t_ is the relative response at time *t* and *R*_0_ is the relative response at the starting time *t*_0_. The association rate constants were calculated from the measured *k*_diss_ according to the equation *R*_t_ = *R*_eq_[1−*e* ^(*k*^_diss_ *^C+k^*_diss_^)(*t−t*0)^]. Here, *R*_eq_ is the steady-state response level and *C* is the molar concentration of the analyte. The dissociation constant (K_D_) were calculated by dividing *k*_diss_ by *k*_ass_.

### Protein structure accession number

Atomic coordinates and structure factors have been deposited in RCS Protein Data Bank (PDB) with accession code 3GXV.

## Results

### NTD of *Hp*DnaB exists as a dimer in solution

The NTD (1–121 residues) of *Hp*DnaB was expressed and purified as described in Materials and Methods. The elution profile of size exclusion column shows that the protein exists in a dimeric form (Figure 1A). The protein was isolated after gel filtration to homogeneity as estimated by SDS PAGE (Figure 1B). Rhombohedral crystals (0.15×0.15×0.1 mm) grew overnight against a crystallization well solution containing 0.5 ml reservoir solution containing 5% PEG 400, 150 mM ammonium sulphate and 100 mM Bis-Tris pH 5.5 (Figure 1C). Unfortunately, NTDL (1–144 residues) did not form crystals under our experimental conditions.

### Data collection and structure determination

Initial attempts for crystallization yielded small, rhombohedral shaped crystals that appeared overnight at 4°C. Several crystals were tried for data collection and few crystals diffracted with pseudomerohedral twinning were processed in tetragonal space group but refinement did not yield satisfactory R-factors (data not shown). Following this, a wide variety of crystallization conditions explored. Fortunately, the crystals appeared in the conditions mentioned in Materials and Methods, which diffracted to 2.2 Å resolution in orthorhombic space group without twinning. The best structure solution was obtained with recently released structure of NTD of *Mycobacterium tuberculosis* helicase (Nt*Mtb*DnaB, PDB iD: 2r5u), with two molecules in one asymmetric unit. The data processing and refinement statistics are in Table 1 and a portion of 2F_o_-F_c_ electron density showed in Figure 2B.

**Table 1:** Data collection and refinement statistics.

**Crystallographic data**	
X-ray Source	BESSY
Wavelength (Å)	0.918
Space Group	P2_1_2_1_2_1_
Unit-cell parameters (Å)	
*a*	71.83
*b*	78.15
*c*	108.00
Vm (Mattews volume, Å^3^ Da^−1^)	5.42
Resolution range (Å)	20–2.2(2.3–2.2) a
R_sym_ (%)	9.5(57.5) a
Completeness (%)	96.8 (88.6) a
Total No. of observations	162496(6637) a
No. of unique observations	30563(1797) a
Redundancy	5.32(3.69) a
Average *I/σ (I)*	9.78(2.35) a
**Refinement**	
Resolution	50.0–2.2
R-factor (%)	24(28) a
Free R-factor (%)	28(32) a
Mean B factor	65.85
Number of atoms	2643
Protein/water/other	2525/118
**RMS deviations**	
Bonds (Å)	0.007
Bond angles	1.3
Dihedral angles	19.4
Improper angles	0.9
Cross validated error	0.34

a Numbers in parentheses are for the last resolution shell.

### Overall Structure

The NTDs of helicases are relatively less conserved (about 25% identity) (Figure S2) compared to the C-terminal ATPase domain (about 40% identity) and these two domains are connected by a much less conserved linker region. We determined the structure of NTD of *Hp*DnaB which is highly helical with a compact head (N-globe) and a long helix (H7) at the C-terminal end (Figure 2A). The asymmetric unit consists of two complete monomers and two short peptide fragments. Interestingly, the two complete monomers form a dimer which is stabilized by the two short peptides and it eventually appears as a heterotetramer (Figure 2C). In a dimer, the two monomers are arranged in a head-to-head manner where the C-terminal helices are extended parallel to each other. The two short peptides form helices, which are arranged in an anti-parallel fashion with the extended C-terminal helices (H7) resulting to the formation of a four helix bundle. The head-to-head dimer organization as seen in NTD of *Hp*DnaB is different from other homologous structures known so far. The structure leads to greater understanding of the formation of a stable dimer through linker which is an important event for hexamerisation of full length *Hp*DnaB. Also, it reveals surprising details about the linker region of helicase that was earlier reported to be highly flexible and unstructured [37].

Each monomer of NTD consists of seven helices out of which helix1 to helix6 are wrapped to form a globular structure (N-globe) and helix7 extends out from the N-globe. The freely hanging helix7 is highly unstable as it consists of hydrophobic residues on the surface. These hydrophobic residues in the helix7 of two monomers are stabilized by the four helix bundle created all the way through two short peptides which are about 25 residues long helices (Figure 2D). The electron density suggested that these peptide sequences are similar to helix 7 and MALDI (Matrix-Assisted Laser Desorption/Ionization) analysis of crystals confirmed that these are degraded peptides of helix 7 from NTD itself. The protein degradation was further confirmed by SDS-electrophoresis of crystals (Figure S1) and MALDI analysis of small degraded peptide found on the 20% SDS-PAGE. Interestingly, the two short peptides are arranged diagonally to each other and interact with the two parallel helices (C-terminal long helix) from two molecules thus forming a four helix bundle (Figure 2D). This arrangement of four helix bundle allows dimerisation of NTD of *Hp*DnaB. The dimeric interface consists of 29 hydrophobic residues that bury 3410.56 Å^2^ of solvent accessible surface area.

### Comparison with other structures

The comparison of helicase NTD structures from homologous organisms reveals an unusual arrangement in NTD dimers of *Hp*DnaB. Although, the overall structure of the NTD of *Hp*DnaB is similar to NTDs of *Mtb*DnaB, *Bst*DnaB, *Taq*DnaB and *Ec*DnaB helicase structure with root-mean-square deviation (RMSD) of 1.42 Å (102 Cα atoms), 1.46 Å (101 Cα atoms), 1.34 Å (93 Cα atoms), 1.45 Å (87 Cα atoms), respectively, even though they share only about 25% sequence identity. An overlay of NTD of *Hp*DnaB on *Mtb*DnaB (Figure 2E) confirms the propensity of helix7 to be stabilized by forming a helical hairpin. The helix 7 in NTD of *Hp*DnaB is stabilized by the short auto-degraded helices. The overlaying of *Hp*DnaB on NTD of *Ec*DnaB (Figure 2F) shows that the globular head is compact in both organisms with slight differences in the orientation of secondary structures. As the crystal structure of NTD of *Ec*DnaB lacks the linker region, it does not throw any light on the dimeric organization through linker. Whereas in the solution structure of a little longer construct of NTD of *Ec*DnaB where the linker residues are present, shows a highly disordered linker region [37]. Consequently, the similarity in overall structure is due to the globular domain organization but dimerisation is dependent on the structural assembly and length of linker region that differs in the crystal structures of different NTDs from different organisms.

It has been observed that the NTD of *Hp*DnaB exists as dimers like NTD of *Mtb*DnaB in solution [22], whereas the NTD of EcDnaB exists in monomeric form [37]. However, in crystals, both the NTD of *Hp*DnaB and *Ec*DnaB form dimers, whereas *Mtb*DnaB is observed as a hexamer. Here, *E.coli* NTD refers to the globular domain only and *H. pylori* NTD consists of the globular domain along with first helix from linker. However, in *Mtb*DnaB the NTD includes globular domain and two helices from linker region, where these two helices form α-helical hairpin structure. The architectural details of these three structures allow us to understand the differences in dimer formation. The dimers formed by NTD of *Ec*DnaB in crystal structure show interactions through the globular domain alone. While in the solution structure of longer NTD of *Ec*DnaB, the linker does not form helical hairpin rather it is unstructured [37]. In the NTD structure of *Mtb*DnaB, the dimers are formed in a head-to-tail fashion through the four helix bundle, where the helix hairpins from both the molecules are arranged in a head-to-toe manner (Figure S3). However, the NTD of *Hp*DnaB construct has a half of the helix hairpin (Helix 7) which does not allow the formation of four helix bundle as seen in the structure of *Mtb*DnaB. Stability in NTD of *Hp*DnaB is achieved by forming head to head dimers with four helix bundles at the dimer interface along with short peptides. This clearly shows that the helix hairpin formation in the linker region is highly preferred to enable a biological dimer assembly. The sequence analysis of linker region (helix 7 to 8) reveals, the i(a), i+3(d) and i+4(e) residues in heptad repeats are hydrophobic in nature, which is essential and responsible for four helix bundle formation and this pattern is conserved among different helicases (Figure S4). Although, the linker region is least conserved among the helicases but it is observed for the first time that the linker sequences (helix 7 and 8) have intrinsic property to form four helix bundles as shown by sequence analysis.

### Binding of Primase stimulates helicase activity

**Figure 4:**
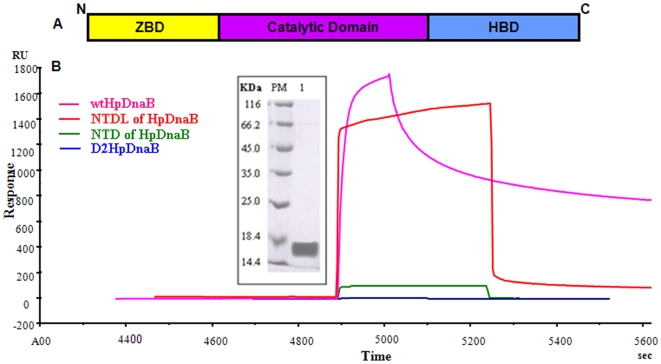
Schematic of *Hp*DnaG primase and helicase-primase interactions in *H.pylori*. (A) A diagram showing the domain organization of *Hp*DnaG primase. The N-terminus domain is the Zinc Binding Domain (ZBD, yellow) linked through the catalytic domain (magenta) to the C-terminal Helicase Binding Domain (HBD, blue). (B) Surface Plasmon Resonance sensogram obtained after injection of wt*Hp*DnaB, D2*Hp*DnaB, NTDL and NTD of *Hp*DnaB on immobilized CTD of *Hp*DnaG. *INSET* shows the SDS-PAGE of purified CTD of *Hp*DnaG (lane1) along with protein marker (PM).

To study the *Hp*DnaG-*Hp*DnaB interactions, we had cloned, expressed and purified a putative homolog of DnaG in *H. pylori* (ORF, *Hp*0012, *Hp* strain 26695) for the very first time. The purity and homogeneity of the protein was further checked by SDS-PAGE analysis as shown in (Figure 3A). The schematic diagram displaying the domain organization in *Hp*DnaG is shown in Figure 4A. The C-terminal domain of *Hp*DnaG binds to the dimeric NTD of DnaB helicase as seen in *Bst*DnaB-DnaG complex structure. Accordingly, we have purified C-terminal primase domain for interaction studies with *Hp*DnaB using surface plasmon resonance (SPR) (Figure 4B).

The biochemical studies of *Bst*DnaB-DnaG interactions demonstrate that the residues I_119_ and I_125_ of DnaB establish the foundation for binding of two proteins [38]. These residues fall in the longer helix of helical hairpin of the linker region (Figure S2) whose counterpart is present in the NTD of *Hp*DnaB used here for crystallization studies. As it is evident from the structure, we were interested to determine whether the unusual dimers of NTD of *Hp*DnaB provides similar platform for binding to CTD of *Hp*DnaG. The sensograms of wt*Hp*DnaB, D2HpDnaB, NTDL and NTD of *Hp*DnaB are shown in Fig. 4B. Sensograms of wt*Hp*DnaB showed a rapid increase in SPR signal during the association phase corresponding to a fast on-rate (K_on_), and a gradual reduction in SPR signal during dissociation phase corresponding to a slow off-rate (K_off_), suggesting a higher affinity of wt*Hp*DnaB to CTD of *Hp*DnaG (K_D_ = 2.25 µM). On the other hand, a comparatively similar SPR signal accompanied by a faster K_on_ and a faster K_off_ was observed for NTDL of *Hp*DnaB indicating a relatively less affinity towards CTD of *Hp*DnaG (K_D_ = 0.11 µM). A relatively faster K_on_ and K_off_ was observed for NTD of *Hp*DnaB along with a very less SPR signal, indicating that the NTD binds with very less affinity to *Hp*DnaG. The NTD deleted construct (D2*Hp*DnaB) does not binds to CTD of *Hp*DnaG showing the binding takes place through NTD of *Hp*DnaB. Surprisingly, we observed that the NTD of *Hp*DnaB shows very weak binding to *Hp*DnaG primase compared to NTDL and HpDnaB, suggesting that N-terminal domain needs to be in a proper conformation for interaction with *Hp*DnaG primase.

These results enhanced our curiosity to determine whether *Hp*DnaG stimulates the *Hp*DnaB helicase activity which is the hallmark of this interaction at the replication fork. For this purpose, the release of radiolabeled unwound oligo from an annealed M13 based helicase substrate was followed in the presence of fixed amount of *Hp*DnaB and increasing amount of *Hp*DnaG as described in Materials and Methods. We find that at low concentration of *Hp*DnaB (20 ng), the release of unwound oligo from the helicase substrate is very weak whereas increasing amount of *Hp*DnaG results in significant stimulation of *Hp*DnaB helicase activity (Figure 3B). *Hp*DnaG by itself does not show any helicase activity under the same experimental conditions. These results clearly suggest that *Hp*DnaB helicase activity is stimulated by *Hp*DnaG and CTD of *Hp*DnaG (results not shown) *in vitro*.

### Interactions in Helicase-Primase complex

The crystal structure of *Bst*DnaB in complex with CTD of primase [20] was taken as the basic model to understand the helicase-primase interactions. The residues from NTD of DnaB helicase, which participate in primase interactions, come from three different parts of the primary structure (Figure S2). Two NTDs in the hexameric state of DnaB helicase organize to form a binding surface for primase in three-dimensional space with these three regions. These regions are relatively better conserved in NTD of *Hp*DnaB in contrast with the rest of the N-terminal region (Figure S2). The critical primase interacting residues from helicase are partially conserved. The differential recognition or binding affinity with primase must be obtained by certain variations in helicase and these variations may be compensated by low homology of the C-terminal domain of primase. For example there are few differences in these three regions, like 64 (L), 65(V/I) and 100(I) which are conserved hydrophobic residues in *Bst*DnaB, *Ec*DnaB, *Mtb*DnaB and *Taq*DnaB helicases, while these residues are replaced by hydrophilic residues (E, N & E) in *Hp*DnaB. Similarly in primase, three different parts of the sequence come closer in three-dimensional space forming a surface that interacts with NTDs of the two helicases. The primase C-terminal domain has much lower sequence homology compared to the rest of the primase domain or even its interacting counterpart helicase NTD (Figure S5). Among the four primase sequences, the *E.coli* DnaG exhibits high diversity and even has an insertion in one of the binding regions. This may explain the weak binding affinity between the *E.coli* primase and its helicase.

## Discussion

The NTD of *Hp*DnaB forms dimers in solution state and in the crystal structure. The NTDs of *Hp*DnaB and *Ec*DnaB exist as dimers in crystal structures while NTD of *Mtb*DnaB is found in the hexameric state. These conformational differences may be primarily due to the relative length of the N-terminal domain construct. The presence of helical hairpin keeps the NTD of *Mtb*DnaB in dimeric state by forming the four helix bundle in head to tail manner, and tertiary interactions of N-terminal domain induce these dimers to assemble as hexamer in crystal state. Helix 7 and 8 belongs to the hairpin forming region of the linker, which is crucial in the positioning of N-terminal lobe during hexamerization of helicase. The degradation of Helix 7 and formation of four helix bundle in our NTD structure and sequence analysis of this region clearly shows that this region has very high tendency to form four helix bundles through hairpin structure to stabilize the hydrophobic residues. The assembly of NTD of *Hp*DnaB through four helix bundle formation and the propensity of helix 7 to form a hairpin structure emphasize on the crucial importance of the linker (specifically helix 7 and 8) in the organization, conformation and function of helicases.

**Figure 5:**
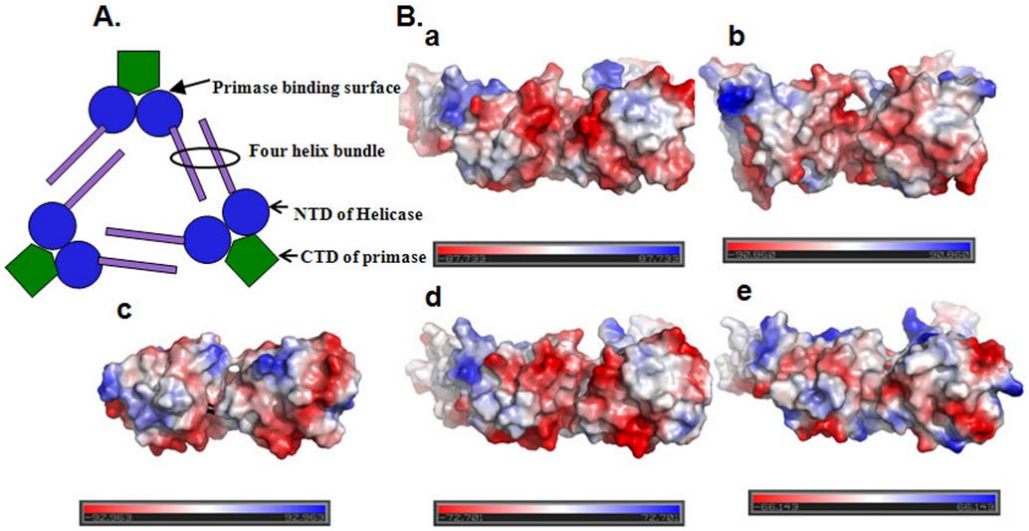
Electrostatic potential surface charge distribution on the primase binding surface of helicases. (A) A schematic representation of helicase-primase complex to depict the primase binding surface on helicase and the organization of two NTDs in a hexamer to facilitate primase binding. (B) The electrostatic potential surface of NTDs of (a) *Bst*DnaB, (b) *Hp*DnaB, (c) *Ec*DnaB, (d) *Taq*DnaB and (e) *Mtb*DnaB. The NTD dimer of *Bst*DnaB-DnaG complex structure was taken as template to generate the model for respective dimers of other helicases. Negative, neutral and positive electrostatic potentials are displayed in red, white and blue, respectively.

The helical hairpin structure present between the NTD and CTD of helicase plays a critical role in organizing the two NTDs in specific dimeric conformational state by forming four helix bundles. The quaternary interactions of the N-terminal and C-terminal domain stabilize three dimers in the hexameric state (Figure 5A), which is essential for primase binding. The binding studies show that the CTD of primase binds with a high affinity to the full length helicase and NTDL; poorly with NTD of *Hp*DnaB alone. In the helicase-primase structure of *Bst*DnaB, the two globular heads of NTDs in a hexamer faces one direction to interact with primase whereas in the present structure of NTD of *Hp*DnaB the globular heads are arranged opposite to each other. We presume in NTDL, the helix 7 and 8 from linker region form hairpin which is involved in dimer formation by head to tail arrangement that in turn leads to a hexameric organization, similar to *Mtb*DnaB and that may be responsible for better primase binding. It indicates that the conformational state of NTD of helicase plays a pivotal role in the stability of helicase-primase interactions.

Despite very low sequence homology, the overall globular domain structure of NTD of *Hp*DnaB is similar to NTDs' of *Ec*DnaB, *Mtb*DnaB, *Bst*DnaB and *Taq*DnaB. Interestingly, the electrostatic potential surface charge distribution on the primase binding surface of helicases show major striking differences in different organisms which may be responsible for the differences in binding stability of DnaB-DnaG complex (Figure 5B). The primase binding surface of NTD of *Bst*DnaB is exclusively covered by negatively charged side chains at center that must be aiding a strong binding with the CTD of *Bst*DnaG, which is rich in positively charged side chains on the of helicase binding surface. Whereas in *E. coli* primase binding surface of NTD, the negative charge distribution is very low compared to NTD of *Bst*DnaB, this may be responsible for a transient helicase-primase binding. The primase binding surface of NTD of *Hp*DnaB shows dense negative charge distribution similar to NTD of *Bst*DnaB, which may be responsible for strong *Hp*DnaB-*Hp*DnaG binding. These observations suggest that the helicase and primase proteins display charge dependent interactions.

In conclusion, the structural organization of linker region and binding stability of DnaB-DnaG complex as indicated by surface charge distribution of primase binding surface on helicase shows clearly that *Hp*DnaB differs greatly from *Ec*DnaB despite both belong to gram negative bacteria. Interestingly, the NTD of *Hp*DnaB resembles more with NTDs of *Mtb*DnaB and *Bst*DnaB helicase of gram positive bacteria. The structure of full length DnaB helicase can explain the self-loading phenomenon. Future work of determining the structure of full length *Hp*DnaB/NTDL with primase/primase domain complex will through more light on helicase-primase interactions in *H.pylori* that may lead to more general understanding of helicase-primase interactions.

## Supporting Information

Figure S1 The MALDI profile of degraded fragment of NTD of HpDnaB. The fragmentation pattern of a peptide m/z = 1895.75 is clearly in agreement with the C-terminal sequence NTIREQALEHHHHHH. Inset shows 20% SDS PAGE with lane1, crystal; lane2, Drop without crystal; PM, Protein marker, clearly showing small fragment along with NTD of HpDnaB in crystal. Lane 2, mother liquor shows protein band corresponding to NTD of HpDnaB and some degraded protein.
(1.35 MB TIF)

Figure S2 Sequence alignment of NTD of DnaB helicase. Multiple sequence alignment of NTD of DnaB from several species (Hp, H. pylori, Bst, B. stearothermohillus; Taq, T. aquaticus; Mtb, M. tuberculosis; Ec, E.coli) are aligned using ClustalW. The (.), (:) and (*) symbols at the bottom of alignment represents the semi conserved, conserved and identical residues in the column. The secondary structures are shown as seen in NTD of HpDnaB. Filled circles represent the residues taking part in helicase-primase interactions as seen in helicase-primase complex structure [20] and filled squares represent the crucial residues essential for maintaining the helicase-primase interactions from biochemical studies in B.stearothermophilus [38].
(1.32 MB TIF)

Figure S3 Differences in dimer organization. Dimer organization of (A) NTD from HpDnaB and (B) NTD from MtbDnaB.
(0.64 MB TIF)

Figure S4 Sequence alignment of helical hairpin region of DnaB helicase showing the heptad repeat leading to four helix bundle formation. Multiple sequence alignment of helical hairpin region of DnaB from several species (Hp, H. pylori, Bst, B. stearothermohillus; Taq, T. aquaticus; Mtb, M. tuberculosis; Ec, E.coli) are aligned using ClustalW. The (.), (:) and (*) symbols at the bottom of alignment represents the semi conserved, conserved and identical residues in the column. The heptad repeat is labeled as “abcdefg” on the top. The i(a), i+3(d) (highlighted with green color) and i+4(e) (highlighted with cyan color) positions are well conserved by hydrophobic residues in helix 8 and C-terminal side of the helix 7 indicating that these induce the four helix bundle formation. In the beginning of helix 7 the i(a) and i+3(d) are conserved with hydrophobic residues indicating that they prefer coiled-coil formation.
(0.47 MB TIF

Figure S5 Sequence alignment of Helicase Binding Domain (HBD) of DnaG primase Multiple sequence alignment of C-terminal domain (HBD) of DnaG from several species (Hp, H. pylori, Bst, B.stearothermophilus; Bs, B. subtilis; Ec, E.coli) are aligned using ClustalW [39]. The (.), (:) and (*) symbols at the bottom of alignment represents the semi conserved, conserved and identical residues in the column. Filled circles represent the helicase interacting residues as seen in B.stearothermophilus helicase-primase complex [20].
(0.93 MB TIF)
