# Supplementary figures and images for: Three-Dimensional Structure of N-Terminal Domain of DnaB Helicase and Helicase-Primase Interactions in Helicobacter pylori
Source: PLoS One. 2009 Oct 20;4(10):e7515. doi: 10.1371/journal.pone.0007515 (PMC2761005; doi:10.1371/journal.pone.0007515)

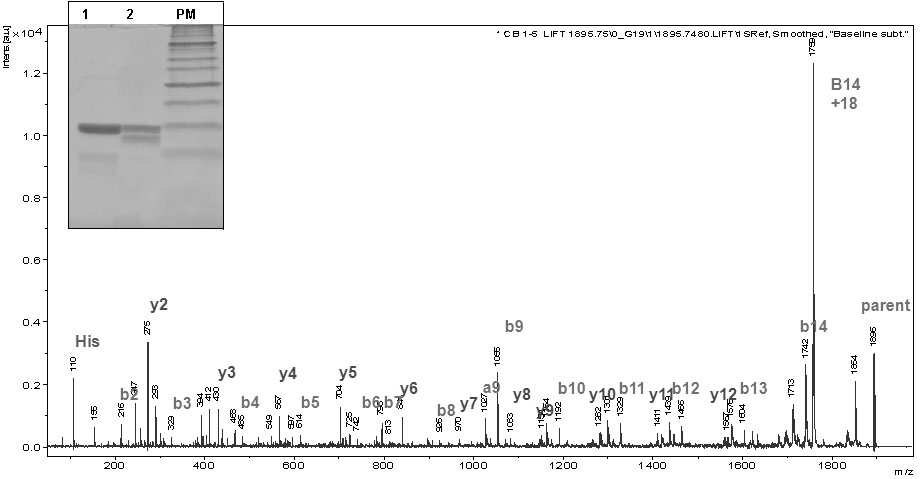

Supplement: Figure S1 — The MALDI profile of degraded fragment of NTD of HpDnaB. The fragmentation pattern of a peptide m/z = 1895.75 is clearly in agreement with the C-terminal sequence NTIREQALEHHHHHH. Inset shows 20% SDS PAGE with lane1, crystal; lane2, Drop without crystal; PM, Protein marker, clearly showing small fragment along with NTD of HpDnaB in crystal. Lane 2, mother liquor shows protein band corresponding to NTD of HpDnaB and some degraded protein. (1.35 MB TIF) [file pone.0007515.s001.tif]

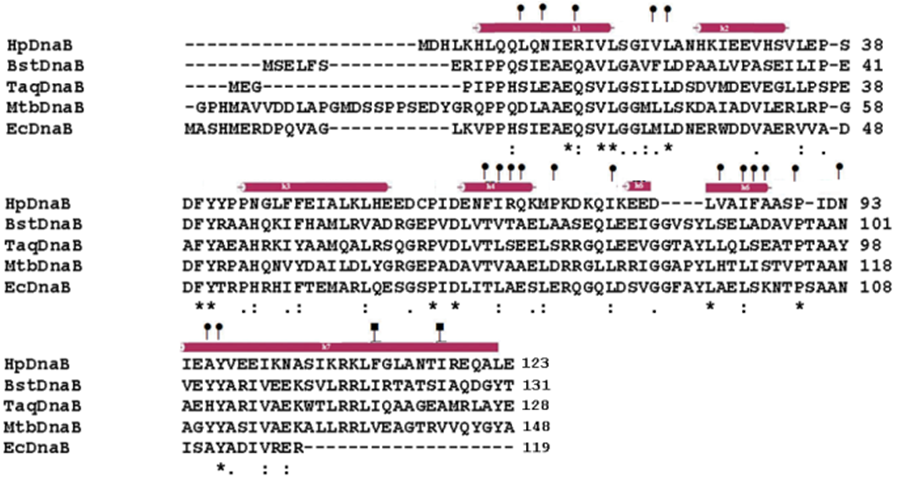

Supplement: Figure S2 — Sequence alignment of NTD of DnaB helicase. Multiple sequence alignment of NTD of DnaB from several species (Hp, H. pylori, Bst, B. stearothermohillus; Taq, T. aquaticus; Mtb, M. tuberculosis; Ec, E.coli) are aligned using ClustalW. The (.), (:) and (*) symbols at the bottom of alignment represents the semi conserved, conserved and identical residues in the column. The secondary structures are shown as seen in NTD of HpDnaB. Filled circles represent the residues taking part in helicase-primase interactions as seen in helicase-primase complex structure [20] and filled squares represent the crucial residues essential for maintaining the helicase-primase interactions from biochemical studies in B.stearothermophilus [38]. (1.32 MB TIF) [file pone.0007515.s002.tif]

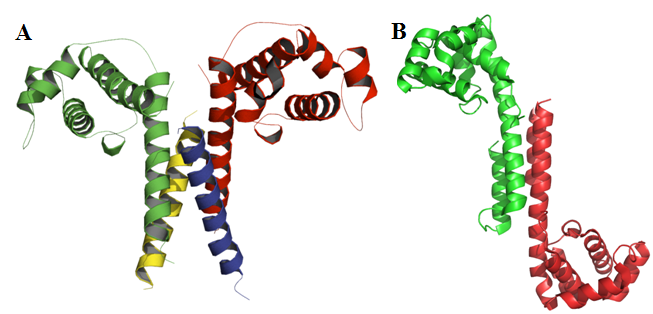

Supplement: Figure S3 — Differences in dimer organization. Dimer organization of (A) NTD from HpDnaB and (B) NTD from MtbDnaB. (0.64 MB TIF) [file pone.0007515.s003.tif]

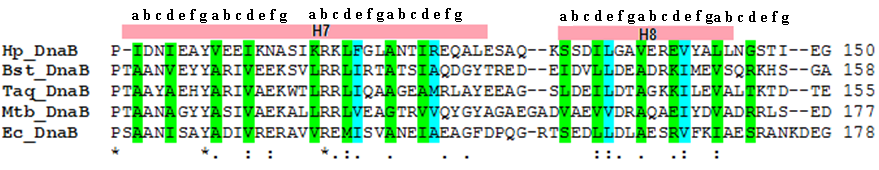

Supplement: Figure S4 — Sequence alignment of helical hairpin region of DnaB helicase showing the heptad repeat leading to four helix bundle formation. Multiple sequence alignment of helical hairpin region of DnaB from several species (Hp, H. pylori, Bst, B. stearothermohillus; Taq, T. aquaticus; Mtb, M. tuberculosis; Ec, E.coli) are aligned using ClustalW. The (.), (:) and (*) symbols at the bottom of alignment represents the semi conserved, conserved and identical residues in the column. The heptad repeat is labeled as “abcdefg” on the top. The i(a), i+3(d) (highlighted with green color) and i+4(e) (highlighted with cyan color) positions are well conserved by hydrophobic residues in helix 8 and C-terminal side of the helix 7 indicating that these induce the four helix bundle formation. In the beginning of helix 7 the i(a) and i+3(d) are conserved with hydrophobic residues indicating that they prefer coiled-coil formation. (0.47 MB TIF [file pone.0007515.s004.tif]

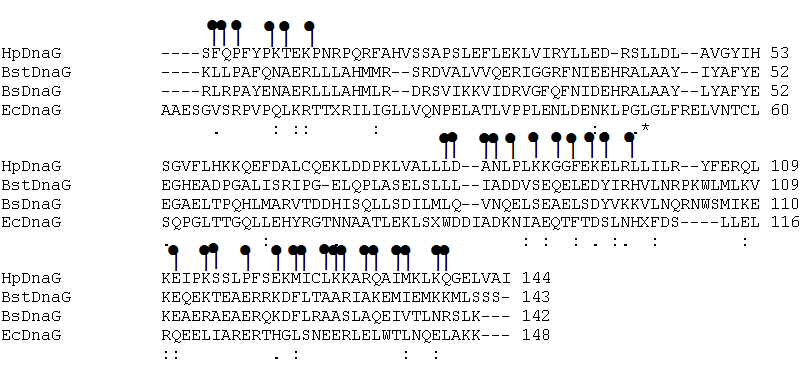

Supplement: Figure S5 — Sequence alignment of Helicase Binding Domain (HBD) of DnaG primase Multiple sequence alignment of C-terminal domain (HBD) of DnaG from several species (Hp, H. pylori, Bst, B.stearothermophilus; Bs, B. subtilis; Ec, E.coli) are aligned using ClustalW [39]. The (.), (:) and (*) symbols at the bottom of alignment represents the semi conserved, conserved and identical residues in the column. Filled circles represent the helicase interacting residues as seen in B.stearothermophilus helicase-primase complex [20]. (0.93 MB TIF) [file pone.0007515.s005.tif]
